# Taurine protects against lung damage following limb ischemia reperfusion in the rat by attenuating endoplasmic reticulum stress-induced apoptosis

**DOI:** 10.3109/17453671003587085

**Published:** 2010-04-06

**Authors:** Xiuli Men, Shuying Han, Junling Gao, Guofu Cao, Lianyuan Zhang, Hong Yu, Hua Lu, Jianyi Pu

**Affiliations:** ^1^Department of Pathophysiology; ^2^Department of Pharmacology; ^3^Histology and Embryology, North China Coal Medical College; ^4^The Affiliated Hospital, North China Coal Medical College, TangshanChina

## Abstract

**Background and purpose** CHOP is a C/EBP family transcription factor involved in endoplasmic reticulum (ER) stress-mediated apoptosis. Several studies have demonstrated that ischemia reperfusion results in apoptosis. Oxidative stress is central to ischemia reperfusion-induced apoptosis. Taurine protects against lung injury after limb ischemia reperfusion (LIR) through antioxidation. The effects of taurine on ER stress-induced apoptosis have not been well explored, however. We studied the effects of taurine in ER stress-induced apoptosis following LIR.

**Methods** Adult male Sprague-Dawley rats (n = 40) were randomized into 4 groups: (1) a control group, (2) an LIR group, (3) an LIR group treated with taurine, and (4) an LIR group treated with saline. Bilateral hindlimb ischemia was induced by application of a rubber band proximal to the level of the greater trochanters for 4 h. The treatment groups received either taurine (200 mg/kg as a 4% solution in 0.9% saline) or saline alone prior to reperfusion. Following 4h of reperfusion, blood oxygen was analyzed. The animals were killed and plasma and lung tissue were harvested for evaluation.

**Results** Taurine statistically significantly attenuated lung injury following LIR, as shown by reduced malondialdehyde content, reduced cell apoptosis, and expression of activating transcription factor 4 (ATF4), X-box binding protein 1 (XBP1), and transcriptional activators of the CHOP gene. Furthermore, partial pressure values of oxygen in arterial blood and the activities of superoxide dismutase and catalase were higher in the taurine pretreatment group than in the group of rats that underwent LIR alone.

**Interpretation** Our results suggest that taurine attenuates endoplasmic reticulum stress-induced apoptosis in the lungs of rats after limb ischemia reperfusion.

## Introduction

In recent years, there has been increasing evidence that endoplasmic reticulum stress (ERS) plays a crucial role in ischemia reperfusion-induced cell dysfunction (DeGracia and Montie 2004) Most studies have focused on cerebral ischemia, either localized or widespread, using in vitro or in vivo models (Hayashi et al. 2004, 2005, Benavides et al. 2005). There has been little research on ERS in lung injury after limb ischemia reperfusion (LIR). The endoplasmic reticulum (ER) senses oxidative stress and can trigger apoptotic signaling (Szabadkai and Rizzuto 2004, Xu et al. 2005). ERS sensor proteins PERK, IRE1, and ATF6 are activated in the ERS response. ATF4 and XBP1 are induced as cell protection mechanisms. When ER functions are severely impaired, however, CHOP is induced by ATF4 and XBP1, and apoptosis follows.

CHOP, also known as GADD153, is a member of the C/EBP transcription factor family; it forms heterodimers with other C/EBPs (Ron and Habener 1992). CHOP can be induced in response to cellular stress, especially ER stress, and is involved in the ER stress-induced apoptosis pathway (Zinszner et al. 1998).

Taurine is a kind of endogenous free amino acid in tissue and its function may in part be to adjust calcium homeostasis in cells, anti-oxidative stress and anti-inflammatory. It is a good cell protector (Guz et al. 2007). It has been reported that taurine can protect against lung injury following LIR, but the mechanism is unclear (Men et al. 2004).

## Materials and methods

### Model of limb ischemia reperfusion- (LIR-) induced lung injury

40 adult male Sprague-Dawley rats weighing 250–300 g were used. Anesthesia was induced using intraperitoneal sodium pentobarbitone (50 mg/kg). The model for limb ischemia reperfusion was as described previously (Kyriakides et al. 2000). Briefly, ischemia was induced for 4 h followed by 4 h of reperfusion. Bilateral rubber bands (latex O-rings) were applied above the greater trochanter for 4 h. Reperfusion was initiated by removing the bands. Occlusion and reperfusion were confirmed by using a Doppler ultrasound probe placed distal to the site at which the latex O-rings had been applied. Control rats received no such LIR injury. Rats were kept in supine position for the duration of the experiment.The animals were housed in a room at a temperature of 22 ± 2ºC and at 50–60% humidity, with a 12-h day/night cycle. They were fasted for 12 h before the procedure. All animals were killed at the end of the 4 h of reperfusion. All surgical procedures and care were in accordance with institutional guidelines and experiments were performed after receiving approval from the local animal ethics committee (SYXK (JI) 2005-0038).

### Taurine treatment

The rats were randomized into 4 groups of 10. Those in the control group were anesthetized for 4 h and they received neither pretreatment nor injury. Rats in the LIR group underwent 4 h of ischemia and 4 h of reperfusion without any pretreatment. Rats in the taurine pretreatment group had the same treatment as the LIR group, but they also received taurine (Sigma-Aldrich, St. Louis, MO) at 200 mg/kg as a 4% solution in 0.9% saline 30 min before reperfusion, by injection into the lateral tail vein. Rats in the saline pretreatment group received a corresponding volume of drug vehicle (0.9% saline) without taurine. Fluids were administered by slow infusion.

### Biochemical measurements

At the end of the experiment, a polyethylene catheter (0.8 mm outer diameter) was inserted into the carotid artery and blood samples were collected using sterile vented plastic syringes. These were immediately removed and afterwards a small quantity of blood was injected in the distal extremity of the needle to avoid any exchange of gases with the environmental air. Then, partial CO_2_ arterial pressure (PaCO_2_) and partial oxygen arterial pressure (PaO_2_) were measured with a blood gas analyzer (gasometer) Radiometer.

The right lung tissues of each rat were dissected and homogenized with 3 volumes of ice-cold 1.15% KCl. The activities of the antioxidant enzymes superoxide dismutase (SOD) and catalase (CAT), and the levels of malondialdehyde (MDA) were measured in the supernatant, which was obtained by centrifugation at 5,000 rpm.

MDA levels, which reflect the peroxidation rate of lipids in tissue samples, were measured according to Ohkawa et al. (1979). Briefly, the reaction mixture contained 0.1 mL tissue or plasma sample, 0.2 mL of 8.1% sodium dodecyl sulfate (SDS), 1.5 mL of 20% acetic acid, and 1.5 mL of a 0.8% aqueous solution of TBA. The pH of the mixture was adjusted to 3.5, and the final volume was adjusted to 4.0 mL with distilled water; then, 5.0 mL of a mixture of N-butanol and pyridine (15:1, v/v) was added. The mixture was shaken vigorously. After centrifugation at 4,000 rpm for 10 min, the absorbance of the organic layer was measured at 532 nm. The protein concentration of the tissue samples was measured with a Spectronic UV 120 spectrophotometer by the method of Lowry et al. (1951). MDA levels were expressed as nmol/mg protein. SOD activity was measured according to Fridovich (1978). This method employs xanthine and xanthine oxidase to generate superoxide radicals that react with p-iodonitrotetrazlium violet (INT) to form a red formazan dye, which can be measured at 505 nm. Assay medium consisted of 0.01 M phosphate buffer, CAPS (3-cyclohexilamino-1-propanesulfonic acid) buffer solution (50 mM CAPS and 0.94 mM EDTA in a saturated solution of NaOH), pH 10.2, substrate solution (0.05 mM xanthine and 0.025 mM INT), and 80 µL xanthine oxidase. SOD activity was expressed as U/mg protein.

CAT activities were determined by measuring the decrease in hydrogen peroxide concentration at 230 nm by the method of Beutler (1975). The assay mixture consisted of 1 M Tris HCI, 5 mM Na_2_EDTA buffer solution, pH 8.0, 1 M phosphate buffer solution, pH 7.0, and 10 mM H_2_O_2_. CAT activity was expressed as U/mg protein in tissue samples.

### Western blot

All the right lung tissues from each rat were collected and frozen in liquid nitrogen until further analysis. Before analysis, the sample was thawed and homogenized in cold phosphate-buffered saline at 4ºC and transferred to eppendorf tubes. Homogenized tissues were centrifuged at 4,100 g for 30 min at 4ºC. Then the supernatants were collected and the concentration of protein was assayed. The protein was extracted in buffer containing 20 mM Tris-HCl, pH 7.4, 2 mM EDTA, 150 µM NaCl, 10 mM NaP, 1% NP-40, and 1 mM phenylmethylsulfonyl fluoride. Total proteins were fractionated by 15% SDS-PAGE. Immunoblotting was performed using CHOP antibody (Santa Cruz Biotechnology Inc., Santa Cruz, CA) as primary antibody with enhanced chemiluminescence (Millipore, Billerica, MA) for detection. β-actin (44 kD) was used as an internal control.

### TUNEL assay

The now widely used TUNEL reaction (terminal deoxynucleotidyl transferase (TdT)-mediated dUTP-biotin nick-end labeling) was introduced by Gavrieli et al. in 1992 for detection of apoptosis in tissue sections.

Right lung tissue specimens were frozen in OCT compound and sectioned at 5 µm for processing. The terminal deoxynucleotidyl transferase-mediated dUTP nick end-labeling (TUNEL) assay was performed using in situ cell death detection kits according to the recommendations of the manufacturer (Roche) (Stadelmann and Lassmann 2000). The result was viewed by light microscopy and photographed. 3 lung sections from 3 individual animals from each group were selected randomly and analyzed.

### Quantitative real-time RT-PCR analysis

Total RNA was isolated from right lung tissue of each rat using Trizon Reagent. The mRNA was then reverse transcribed by standard procedures. Real-time RT-PCR was used to quantify mRNA levels. Each PCR reaction was carried out in a total volume of 25 µL consisting of 2.5 µL cDNA, 12.5 µL reaction mix (SYBR Green I dye, TOYOBO) and 0.5 µL. In this reaction mixture, each primer should be 0.5µL, and then use free DNase water to make up untill the total volume 25 µL with 25 pmol/L of each oligonucleotide primer. All reactions were performed in an ABI PRISM 7700 Sequence Detection System, in which samples underwent 40 cycles of PCR with an annealing temperature of 55°C. The primers were designed using Primer Premier 5.0 software. For the XBP-1 RNA (NM001004210), the forward primer sequence was GGTATAGCCAGCGAGTGCT and the reverse sequence was TCAGTTGGGAGCCTGATTCT. For the ATF4 RNA (NM024403), the forward primer was GTTGGTCAGTGCCTCAGACA and the reverse primer was CATTCGAAACAGAGCATCGA. For CHOP RNA (NM024134), the forward primer was CCAGCAGAGGTCACAAGCAC and the reverse primer was CGCACTGACCACTCTGTTTC. Rat β-actin RNA was used as an internal control; the forward sequence was GACATCCGTAAAGACCTCTATGCC and the reverse sequence was GAGACACACCTAACCACCGAGATA (173 bp; GI38648901). The value obtained for each specific product was normalized to that for β-actin RNA and expressed as a percentage of the control value.

### Statistics

The results are expressed as mean ± SD. The Kruskall-Wallis test and the Mann-Whitney test were used to evaluate the differences between groups. P-values of < 0.05 were considered to be of statistical significance.

## Results

### Effects of taurine on PaO_2_ and PaCO_2_

**Figure 1.**
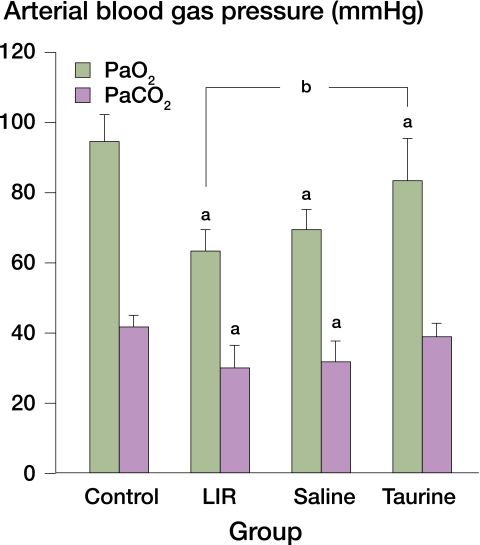
Changes in PaO_2_ and PaCO_2_ in different groups. Data represent mean ± SD of 10 samples. **^a^**p = 0.02 and **^b^**p = 0.03.

There were some changes in PaO_2_ and PaCO_2_ among the 4 groups. Compared with that in the control group, PaO_2_ and PaCO_2_ decreased in the LIR group (Mann-Whitney (M-W) test, p = 0.02). Taurine treatment can increase PaO_2_ (M-W test, p=0.03) and PaCO_2_ (M-W test. p=0.08), but the change in PaCO_2_ was not statistically significant (Figure 1).

### Effects of taurine on SOD, CAT, and MDA in lung tissues

The highest MDA levels in lung tissues occurred after limb ischemia reperfusion for 4 h (LIR group) (M-W test, p = 0.005), and this increase was prevented by pretreatment with taurine. The natural antioxidant enzymes of mammalian cells, SOD and CAT, were also reduced in the LIR group compared to those in control group (M-W test, p = 0.03 and p = 0.02). However, use of taurine before the reperfusion period prevented enzyme levels from falling (Table).

**Table:** SOD, CAT, and MDA content of lung tissue in different groups

Experimental groups	Number of rats	SOD U/mg prot	CAT U/mg prot	MDA nmol/mg prot
Control	10	1.85 (0.25)	1.92 (0.41)	101 (17.3)
LIR	10	0.69 (0.17) **^a^**	1.13 (0.35) **^a^**	151 (19.4) **^a^**
Saline	10	0.73 (0.19) **^a^**	1.21 (0.28) **^a^**	143 (11.2) **^a^**
Taurine	10	1.87 (0.49) **^b^**	2.05 (0.46) **^b^**	122 (12.8) **^b^**
**^a^**p < 0.05 vs control group				
**^b^**p < 0.05 vs LIR group				

### Taurine downregulated the expression of CHOP protein in lung tissues

**Figure 2.**
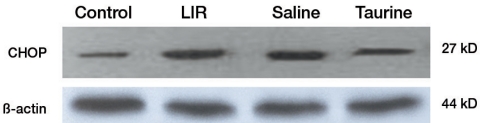
Expression of CHOP protein in the lungs of different groups by western blot.

The expression of CHOP in the lungs of rats in the different groups was detected by western blot. The amount of protein was normalized against the expression of β-actin in the corresponding samples. CHOP protein expression in the LIR group and the saline-pretreated group showed a trend of upregulation compared to that of the control group. Taurine reduced the expression of CHOP protein in lungs after limb ischemia reperfusion, in contrast to the results for the LIR group and saline group (Figure 2).

### Induction of mRNAs for ER stress-related proteins in lung tissue following limb ischemia reperfusion

**Figure 3.**
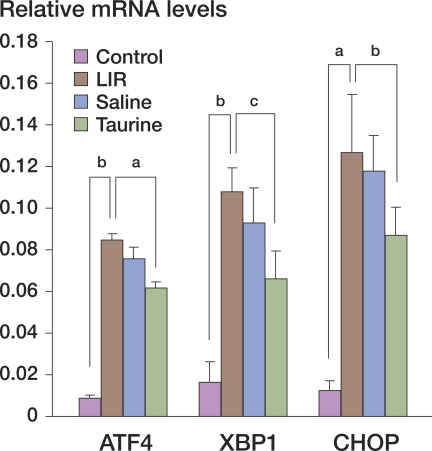
Expression of ATF4, XBP1, and CHOP gene mRNA in different groups as detected by real-time PCR. **^a^**p = 0.03, **^b^**p = 0.03, and **^c^**p = 0.04.

Real-time PCR showed that XBP-1, ATF4, and CHOP mRNA levels in lung tissues of LIR rats drastically increased relative to those of control rats (M-W test, p = 0.027, p = 0.03, and p = 0.02, respectively). Taurine pretreatment downregulated the expression of XBP-1, ATF4, and CHOP mRNA in lung tissues following limb ischemia reperfusion (M-W test, p = 0.017, p = 0.04, and p = 0.03, respectively) (Figure 3).

### Taurine markedly reduced apoptosis in lungs following limb ischemia reperfusion

**Figure 4.**
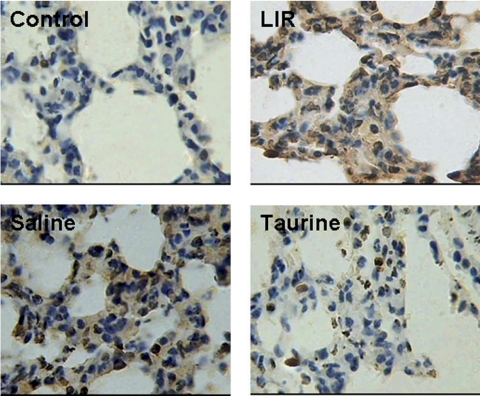
Effect of taurine on apoptosis: TUNEL staining for apoptosis in lungs from different groups.

Apoptosis was detected in each group using TUNEL staining. Limb ischemia reperfusion resulted in apoptosis in the lungs, which was reduced by taurine administration. The protective effect of taurine was accompanied by reduced expression of the UPR-specific proapoptotic protein CHOP and its upstream transcription factors ATF4 and XBP-1 (Figure 4).

## Discussion

It is known that ischemia reperfusion injury promotes ER stress. There is considerable evidence that reactive oxygen species (ROS) play an important role in ischemia reperfusion-induced cell injury or death. Recently, ER stress-mediated apoptosis was reported to be involved in the pathogeneses of several diseases, including diabetes mellitus, neurodegenerative diseases, and ischemia reperfusion (Milhavet et al. 2002, Oyadomari et al. 2002, Yung et al. 2007). CHOP is involved in the ER stress-mediated apoptosis pathway. Yet, little is known about the involvement of ER stress and CHOP-mediated apoptosis in lung injury after limb ischemia reperfusion. Because lung epithelial cells secrete a large amount of proteins such as surfactants, it can be speculated that these cells are prone to ER stress.

Oxidative stress is central to ischemia reperfusion injury. The role of the endoplasmic reticulum (ER) in this process is uncertain. In ER signaling, PERK-ATF4-CHOP and IRE-XBP1-CHOP are two pathways that determine the fate of cells under stress.

Previous studies have shown that ER stress-mediated apoptosis and CHOP-mediated apoptosis are involved in the pathogenesis of hereditary diabetes mellitus in mice caused by a point mutation in the insulin gene, and ischemia-induced neuronal cell death in vivo (Oyadomari et al. 2002).

The C/EBP family protein CHOP is a transcription factor, the expression of which is induced during ER stress. It participates in ER-mediated apoptosis. Downregulation of CHOP activity compromises cell viability, and cells lacking CHOP are significantly protected from the lethal consequences of ER stress (Zinszner et al. 1998).

We have reported that taurine protects against apoptosis in lungs after limb ischemia reperfusion in rats (Men et al. 2004). The mechanism of the protective effect of taurine seems at least to depend on antioxidation. There is increasing evidence that ER stress plays a crucial role in ischemia reperfusion-induced cell dysfunction (DeGracia and Montie 2004).

In the present study, TUNEL staining results showed that apoptosis in the lung tissue of the taurine group rats decreased, in contrast to the results using LIR group rats. Necrotic cells are usually stained very faintly in TdT-based assays, however (Stadelmann and Lassmann 2000). The question of whether apoptosis is positive or negative with respect to the pathogenesis of acute lung injury remains unanswered. Matute-Bello and Martin (2003) suggested that the rate of clearance of apoptotic neutrophils is associated with the resolution of acute inflammation in the lung. In our experiments, LIR-induced apoptosis and PaO2 decreased with LIR group rats, which was prevented in rats pretreated with taurine. MDA levels (an indicator of lipid peroxidation because of oxygen free radicals (OFRs)) detected in the lung tissues of rats were markedly increased in the LIR group and this effect was prevented by the administration of taurine. This finding is in accordance with the notion that the protective effect of taurine against LIR injury is due to its antioxidant effect. Mammalian tissues naturally contain a huge amount of SOD that rapidly converts superoxide anion radicals to H_2_O_2_, which is transformed to H_2_O and O_2_ by CAT. SOD and CAT, which are members of the endogenous antioxidant system, were found to be attenuated in the LIR group, reflecting the over-production of oxygen free radicals (OFRs). The levels of these molecules tended to return to control levels in the presence of taurine, which supports the notion that taurine exerts its antioxidative effects by reducing OFRs.

In order to examine whether taurine reduced apoptosis induced by OFRs related to ER stress, we next examined regulation of the expression of XBP-1, ATF4, and CHOP by taurine. The ATF4 and XBP1 proteins are transcriptional enhancers of the CHOP gene. ATF4 mRNA and XBP1 mRNA, which were present in control rats and had increased markedly in the lungs which limbs underwent 4h of ischemia and 4h of reperfusion. When compared with the effects of taurine, the RT-PCR results indicated that ATF4, XBP-1, and CHOP mRNA levels decreased respectively. Moreover, western blot results showed that taurine also attenuated the expression of CHOP protein. Taken together, these results demonstrate that taurine downregulates the levels of ATF4, XBP-1, and CHOP mRNA.

In conclusion, our study has revealed that taurine prevents oxidative stress-induced apoptosis. Moreover, taurine interferes with the UPR, particularly with regard to the downregulation of ATF4 and XBP-1 expression, and subsequently reduces the level of the proapoptotic transcription factor CHOP. Although speculative, our data suggest that a reduction in oxidative stress-induced apoptosis by taurine may be explained at least in part by a decrease in CHOP function. These findings can be used to establish the experimental basis for the use of taurine in prevention and cure of lung injury induced by limb ischemia reperfusion.
